# Interferon Independent Non-Canonical STAT Activation and Virus Induced Inflammation

**DOI:** 10.3390/v10040196

**Published:** 2018-04-14

**Authors:** Yuchen Nan, Chunyan Wu, Yan-Jin Zhang

**Affiliations:** 1 Department of Preventive Veterinary Medicine, College of Veterinary Medicine, Northwest A&F University, Yangling, Shaanxi 712100, China; nanyuchen2015@nwsuaf.edu.cn; 2 Molecular Virology Laboratory, VA-MD College of Veterinary Medicine and Maryland Pathogen Research Institute, University of Maryland, College Park, MD 20742, USA

**Keywords:** Interferons, JAK/STAT signaling, non-canonical STAT activation, viral antagonism, inflammation

## Abstract

Interferons (IFNs) are a group of secreted proteins that play critical roles in antiviral immunity, antitumor activity, activation of cytotoxic T cells, and modulation of host immune responses. IFNs are cytokines, and bind receptors on cell surfaces to trigger signal transduction. The major signaling pathway activated by IFNs is the JAK/STAT (Janus kinase/signal transducer and activator of transcription) pathway, a complex pathway involved in both viral and host survival strategies. On the one hand, viruses have evolved strategies to escape from antiviral host defenses evoked by IFN-activated JAK/STAT signaling. On the other hand, viruses have also evolved to exploit the JAK/STAT pathway to evoke activation of certain STATs that somehow promote viral pathogenesis. In this review, recent progress in our understanding of the virus-induced IFN-independent STAT signaling and its potential roles in viral induced inflammation and pathogenesis are summarized in detail, and perspectives are provided.

## 1. Introduction

Interferons (IFNs) are a group of secreted proteins that play key roles in host antiviral immunity. IFNs are typically induced by the activation of host pattern-recognition receptors (PRRs), mainly retinoic acid-inducible gene 1 (RIG-I)-like receptors (RLR) and Toll-like receptors (TLR) during viral infection [1,2]. To date, three types of IFNs (I, II, and III) have been characterized. Type I IFNs, or generally called IFNs (mainly IFN-α/β), compose the largest IFN family [3]. Almost all cell types are capable of producing IFN-α/β; however, plasmacytoid dendritic cells (pDC) are considered as the major cell type for IFN-α production during the course of an viral infection [4,5]. Type II IFNs comprise only IFN-γ [6]. Different from type I IFNs, IFN-γ production is restricted to activated T cells, natural killer cells, and macrophages [6]. Type II IFN plays a major role in establishing cellular immunity; however, it induces expression of a group of genes that respond to type I IFN as well [7,8]. Type III IFNs were the latest IFN family and contain IFN-λ1 to 4 [9,10]. IFN-λ signals through unique receptors, but activates the same pathway as type I IFNs [9,11,12].

Induction of IFNs typically results from activation of host PRRs during virus infection. PRRs mainly include RLRs and TLRs [1,2]. After induction, IFNs stimulate cells via activation of specific signaling pathways, mainly the JAK/STAT (Janus kinase/signal transducer and activator of transcription) pathway [13]. Subsequent cascade events after the triggering of the pathway result in expression of IFN-stimulated genes (ISGs) [14]. Generally, hundreds to thousands ISGs can be upregulated by type I IFNs [15]; ISGs include antiviral effectors that restrict virus replication.

The JAK/STAT pathway was initially characterized based on its role in type I IFN-mediated responses [16]. The STAT proteins have also been shown to be critical for transmission of signals of many diverse membrane receptors, such as cytokine and hormone receptors [17]. The JAK/STAT pathway cascade consists of three major components: a surface receptor, JAK, and downstream STAT proteins [18]. Disruption or dysregulation of JAK/STAT function can result in immune deficiency syndromes and cancers [18].

Notably, viruses antagonize JAK/STAT signaling pathway [19]. Therefore, it has been proposed that JAK/STAT pathway antagonism is a virulence factor that could be exploited as a novel strategy to achieve virus attenuation for modified live virus (MLV) vaccine development [20]. This is consistent with observations that mice lacking intact JAK/STAT signaling (IFN receptor or STAT1 knockout mice) are more susceptible to virus infection than wild-type mice [21,22,23,24]. However, our understanding has grown regarding the JAK/STAT pathway and virus-mediated antagonism. It appears that the interplay between virus and the JAK/STAT pathway is more complicated than expected, with opposing effects existing concurrently instead of mutual exclusion. In this review, several topics are discussed: the discovery of the JAK/STAT pathway, including the canonical JAK/STAT pathway activated by IFNs, tyrosine phosphorylation-independent non-canonical STAT activation, virus-induced serine monophosphorylation of STATs, as well as the role of monophosphorylation of STATs during inflammation triggered by viral infection.

## 2. Canonical and Non-Canonical JAK/STAT Activation

### 2.1. Tyrosine Phosphorylation-Dependent Canonical JAK/STAT Activation

There are three key components in the JAK/STAT pathway: a membrane-bound receptor, JAKs, and STATs [17]. So far, four members have been identified within the JAK family: JAK1, JAK2, JAK3, and tyrosine kinase 2 (TYK2). All JAKs are characterized by a C-terminal catalytic kinase domain, and a related, but enzymatically inactive pseudokinase or kinase-like domain [25]. In addition, five other domains are present within the N-terminal regions of JAKs, and these domains share sequence similarity [17]. All seven JAKs domains are now grouped together for designation as the Janus homology domain (JHD) and are numbered in reverse-order from 7 to 1, from the amino- to the carboxyl-terminal region of JAKs [17]. The pseudokinase domain is thought to regulate JAKs kinase activity by interacting with the kinase domain, which is responsible for JAKs basal activity [26,27].

In mammalian species, there are seven members (encoded by individual gene) of the STAT family: STAT1, 2, 3, 4, 5A, 5B, and 6 [28]. Existence of splicing variants (derived from the same pre-mRNA progenitor) is also confirmed for STAT1, STAT3 [29,30,31], STAT4, and STAT6 [32,33]. These STAT isoforms share a common splicing pattern similar to that of STAT1, which generates shorter STAT forms with incomplete transactivation domains (TADs), and function as negative regulators of STATs [31,33,34]. For STAT5s, proteolytic processing of full length protein generate shorter forms of STAT5s with incomplete TADs, which are similar to splicing pattern of other STATs as well [35]. Meanwhile, bioinformatics analysis suggests a remarkable similarity among STAT genes, with the exception of STAT2 [28]. Moreover, all STATs share a similar structure comprised of the following domains: N-terminal domain (ND), coiled-coil domain (CCD), DNA-binding domain (DBD), linker domain (LD), Src homology 2 (SH2) domain, and TAD [36].

Before activation of the JAK/STAT pathway, JAKs non-covalently associate with the cytoplasmic tails of cytokine receptors, and are functionally inactive. Upon ligand binding, dimerization or oligomerization of specific membrane receptors leads to JAKs apposition and autophosphorylation of tyrosine residues, thereby switching on tyrosine kinase activities of JAKs. Next, the activated JAKs phosphorylate tyrosine residues located in the cytokine-receptor cytoplasmic domains to provide binding sites for STATs SH2 domains, which recruit STATs to interact with JAKs. STATs are then phosphorylated by JAKs on tyrosine residues around amino acid (aa) 700, depending on the length of a particular STAT (750–850 aa in length) [17]. Finally, depending on the ligands binding to the receptors, homologous or heterologous STAT interactions occur immediately after phosphorylation, through reciprocal SH2 interactions that lead to formation of both STAT homodimers or heterodimers, as transcription factor complexes for nuclear translocation [17,37,38].

**Figure 1:**
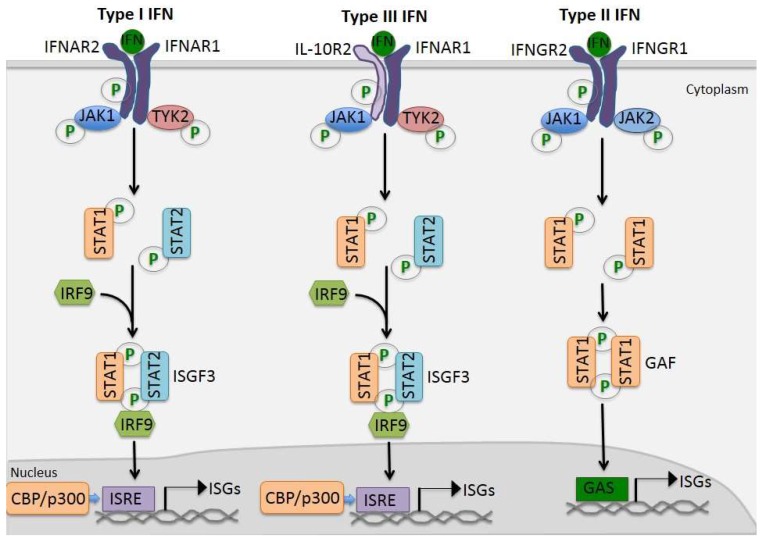
Schematic illustration of Type I, Type II, and Type III Interferon (IFN) signaling.

STAT1 and STAT2 are the major players involved in type I IFN-mediated signaling and form interferon-stimulated gene factor 3 (ISGF3) complex along with IRF9 [38]. Other complexes induced by type I IFNs also include homodimers for STAT1 to STAT6, and heterodimers, including STAT1-STAT3, STAT1-STAT4, STAT1-STAT5, STAT2-STAT3, and STAT5–STAT6 [38]. Type II IFN activates JAK1 and JAK2, resulting in phosphorylation of STAT1 on the tyrosine residue Y701, as observed in type I IFN signaling. However, this event only leads to the formation of STAT1 homodimers, also known as γ-interferon activation factor (GAF), which binds to interferon-γ-activated-sequence (GAS) elements [38]. Karyopherin-α1 (KPNA1) is the essential importin for nuclear transport of phosphorylated STAT1 [39]. The interaction of STAT1 and KPNA1 involves a non-classical nuclear localization signal (NLS) [39,40]. Besides IFN signaling, STATs are also responsible for transducing signals involving several other families of cytokines. Schematic illustration of IFN-mediated JAK/STAT activation was shown in Figure 1.

### 2.2. Tyrosine Unphosphorylated STATs during Non-Canonical STATs Activation

It is believed that phosphorylation of tyrosine resides (around residue 700) in STATs is an essential step for canonical activation of the JAK/STAT pathway induced by cytokines, including IFNs [41]. However, in the absence of tyrosine phosphorylation, STATs still perform unique functions by constantly shuttling between cytoplasmic and nuclear compartments, and are now considered part of non-canonical STATs activation [42]. The STAT3 was the first well-characterized unphosphorylated STAT (U-STAT) in mammalian cells, due to its constant trafficking into the nucleus in the absence of tyrosine phosphorylation [43]. It was demonstrated that aa150 to 162 in the coiled-coil domain are indispensable for U-STAT3 nuclear import [43]. Interestingly, U-STAT3 and tyrosine-phosphorylated STAT3 dimers were found to interact with the same importin-α isoforms, importin-α3 and importin-α6 [43]. Similar to U-STAT3, a series of mutations and deletions has revealed a region within the coiled-coil domain of STAT5A is critical for nuclear import of unphosphorylated STAT5A [44]. Since then, U-STAT1 [45], U-STAT2, and U-STAT5B all have been identified, and have unique functions which differ from their tyrosine-phosphorylated forms [46,47,48]. Therefore, unphosphorylated STAT forms appear to exist for all STATs, and exhibit unique functions that are distinct from their tyrosine phosphorylated forms [49,50,51].

The functions of U-STATs have been extensively investigated for STAT1 and STAT2, since these STATs have been linked to IFN-mediated signaling. After stimulation of the JAK/STAT pathway by high dose IFN treatment (1000 U/mL), tyrosine phosphorylation of STAT1 and STAT2 could be observed within a half hour, but steadily decreased after two hours, and to basal levels by eight hours [52]. This result is also consistent with previous observations of the maximal nuclear accumulation of STAT1, as visualized by GFP-fused proteins two hours after 500 U/mL IFN-β stimulation [53]. However, IFN-activated ISG expression could last far longer than 24 h after high dose IFN treatment. Conversely, if cells were stimulated with low dose IFN-β (5 U/mL), expression of ISGs increased after six hours and persisted after 48 or 72 h, long after tyrosine-phosphorylated STAT1 returned to basal levels [45]. These observations cannot be fully explained by a canonical STAT activation model, requiring tyrosine phosphorylation of STAT as a prerequisite for downstream gene activation [45]. However, when exogenous STAT1 was introduced into cells without IFN-treatment, a subset of IFN-induced genes was upregulated, suggesting that U-STAT1 promoted expression of certain ISGs in the absence of IFN stimulation [45].

Further analysis has indicated that U-STAT1 and U-STAT2, along with IRF9, can support the formation of unphosphorylated ISGF3 (U-ISGF3) [54]. U-ISGF3 formation requires high levels of IRF9, STAT1, and STAT2 in the absence of tyrosine phosphorylation, although U-ISGF3 could be induced by low level IFN-β as well. It has been proposed that phosphorylated ISGF3 first drives a rapid response phase by binding to canonical ISRE, while U-ISGF3 drives a second prolonged response by binding to distinct ISREs with variable flanking sequences that differ from canonical ISREs during the rapid phase [54]. Nonetheless, the exact function of U-ISGF3 still requires further investigation, although available data suggest that U-ISGF3 drives basal expression of ISGs to protect cells against viral infection under homeostatic conditions [45,55,56]. Some U-ISGF3-induced proteins are capable to mediate resistance to DNA damage in many cancers in which U-ISGF3 is overexpressed [45,54]. In addition to forming U-ISGF3, U-STAT2 is constitutively bound to many IFN-activated promoters in the absence of IFN stimulation, contributing to their basal regulation [46]. Meanwhile, STAT2 and IRF9 can form complexes as well, and direct a prolonged ISGF3-like transcriptional response to achieve antiviral activity in the absence of STAT1 [57]. In fact, these results are consistent with previous reports demonstrating that STAT2 mediates innate immunity in the absence of STAT1 [58].

The function of U-STAT3 has been investigated as well, since STAT3 is linked with oncogenesis and IFN-activated responses [59]. When a STAT3-Y705F mutant is overexpressed in STAT3-null cells, some well-known oncoproteins, such as MRAS and MET, are upregulated by U-STAT3, but not by activated STAT3 dimers [59]. These results suggest that U-STAT3 activates gene expression by a novel mechanism distinct from canonical STAT3 dimers [59]. This observation is consistent with recent research demonstrating that U-STAT3, during Interleukin (IL)-6 stimulation, drives a second wave of gene expression that does not respond directly to STAT3 containing phosphorylated tyrosine [60]. Meanwhile, it appears that U-STAT3-responsive genes contain nuclear factor kappa-light-chain-enhancer of activated B cells (NF-κB) response elements that are activated by a novel transcription factor complex formed when U-STAT3 binds to unphosphorylated NF-κB (U-NF-κB). NF-κB response elements come into play after U-STAT3/U-NF-κB trafficking to the nucleus that depends on the presence of the nuclear localization signal (NLS) of STAT3. Once in the nucleus, U-STAT3/U-NF-κB activates a subset of NF-κB-dependent genes [60]. Moreover, the U-STAT3/NF-κB complex appears to activate NF-κB-regulated genes in B-cell neoplasms, and contributes to pathogenesis of those cells as well [61]. These observations are also consistent with another report demonstrating that nuclear U-STAT3 accumulation correlates with a poor prognosis for human glioblastoma [62], suggesting an important role of U-STAT3 in oncogenesis [63]. For IFN-mediated signaling, a recent study suggested that U-STAT3 plays an important role in the IFNs response pathway and showed that 60% of interferon-stimulated genes are STAT3-dependent and 30% are independent of STAT3 tyrosine phosphorylation [49]. In fact, U-STAT3 has been shown to be recruited to promoters of STAT3-regulated ISGs as well. With the exception of U-STAT1 and U-STAT3, tyrosine-unphosphorylated STAT5 (U-STAT5) has been reported to restrain megakaryocytic differentiation and activation of a canonical pSTAT5-driven response that includes regulators of apoptosis and proliferation [48,50].

### 2.3. Serine Monophosphorylation of STATs during Non-Canonical STAT Activation

In addition to tyrosine phosphorylation, STAT serine residues within the C-terminal transactivation domain (TAD) can be phosphorylated as well [41]. Initially, phosphorylation of serine at the TAD was thought to contribute to the attainment of maximal STAT transcriptional activity in addition to tyrosine dependent STAT activation [64]. An earlier in vitro study had suggested that IFN-γ-induced phosphorylation of serine 727 (S727) in STAT1-TAD occurs only on promoter-bound STAT1 [65], which is similar to IL-6-induced S727-phosphorylation of STAT3 [66]. These data suggest that cytokine-induced TAD serine phosphorylation of STATs is accomplished by components of the general transcription machinery that are assembled at the promoter. These hypotheses are further supported by identification of chromatin-associated cyclin-dependent kinase 8 (CDK8) as the kinase responsible for IFN-γ-induced STAT1-Ser727 phosphorylation inside the nucleus [41]. During IFN-γ stimulation, CDK8-mediated STAT1 serine phosphorylation has both positive and negative effects on over 40% of IFN-γ-responsive genes. Moreover, siRNA-mediated silencing of CDK8 renders cells more susceptible to vesicular stomatitis virus (VSV) infection, with a 10-fold higher IFN-γ requirement for efficient protection against VSV [41]. Conversely, a report from another group demonstrated that STAT1-S727 could be phosphorylated by mitogen-activated protein (MAP) kinases as well [67]. A recent study also demonstrated diptoindonesin G-induced extracellular signal–regulated kinases (ERK)-mediated phosphorylation and nuclear translocation of pSTAT1 (S727) in acute myeloid leukemia cells is independent of tyrosine phosphorylation at aa701 of STAT1 [68]. Meanwhile, nuclear translocation of pSTAT1 (S727) promotes specific expression of ISGs, such as Interferon-induced protein with tetratricopeptide repeats (IFIT)3 and The chemokine (C-X-C motif) ligand 1 (CXCL1) [68]. Therefore, monophosphorylation of serine residues of STATs in TAD domains represents a novel non-canonical pathway of STATs activation.

So far, the in vivo function of serine monophosphorylation within the TAD of STATs has been exclusively investigated for STAT1. Previous studies in *Stat1*−/− cells demonstrated the occurrence of STAT1-independent, STAT2-dependent gene expression is a delayed event during the transcriptional response to type I IFNs [57]. Intriguingly, in vivo data from STAT1-Y701F mice demonstrated that the presence of STAT1 (Y701F) partially repressed STAT2/IRF9-dependent, STAT1-independent ISG expression during the late stages of the IFN-β response. Moreover, macrophages obtained from STAT1-Y701F-mutated mice were more susceptible to *Legionella pneumophila* infection than were wild-type macrophages [69]. However, macrophages from STAT1Y701F mice exhibited a modest gain-of-function in antibacterial immunity in comparison with *Stat1*−/− mice upon infection with intracellular microbe *Listeria monocytogenes* [69,70]. Meanwhile, STAT1Y701F mutants partially retained NK cell cytotoxicity compared to the complete loss of that function in *Stat1*−/− mice. However, the NK maturation defect in the STAT1Y701F mice was similar to that observed in *Stat1*−/− mice [69,70]. Conversely, *Stat1-S727A* mice exhibited slightly elevated numbers of mature NK cells (mNKs) in bone marrow, spleen, and blood [71]. Unexpectedly, purified and in vitro-expanded NK cells derived from *Stat1-S727A* mice show significantly higher cytotoxicity against a range of tumor cells [71,72]. Although IFN-mediated signaling has not been completely investigated in these animal models to elucidate the exact role of STAT1-S727, it is possible that phosphorylation of STAT1-S727 plays a role in a cell-specific manner.

Current understanding of tyrosine phosphorylation-independent non-canonical STATs activation remains limited. Previous studies focusing on U-STATs mainly investigated phosphorylation of tyrosine, but rarely examined serine phosphorylation status within the TAD domain at the same time [45,55,73]. Therefore, it is not known if U-STATs or components of U-ISGF3 are completely unphosphorylated at both tyrosine and serine residues, or actually contain phosphorylated serine reside within the TAD domain [74]. Notably, in STAT1-Y701F mice, decreased expression of STAT1-Y701F protein was observed, and impaired U-STAT1-mediated U-ISGF3 signaling as a high level of STAT1 is required for formation of U-ISGF3 [70]. Therefore, the link between U-STATs and serine monophosphorylation of STATs remains elusive, and it is unclear whether they have the same or distinct functions. Further clarification is required to define the role of U-STATs and serine monophosphorylated STATs.

The kinase responsible for monophosphorylation of serine residues of STAT TAD domains in the absence of tyrosine phosphorylation remains elusive so far. Screening of specific CDK8 kinase inhibitors as targeted drugs for cancer therapy has demonstrated that inhibition of CDK8 kinase can result in decreased phosphorylation of STAT1 at S727 in a variety of cancer cells, and phosphorylation of STAT1-S727 could serve as a biomarker of CDK8 kinase activity in vitro and in vivo [75,76,77]. Moreover, when examining TAD serine phosphorylation for other STATs, other reports have demonstrated that CDK5 is responsible for phosphorylation of STAT3 at S727 when T cells were stimulated with Transforming growth factor(TGF)-β and IL-6 during tyrosine phosphorylation of STAT3 [78]. Meanwhile, a higher level of serine monophosphorylation of STAT5 was found in acute myelogenous leukemia (AML), and appears to be CDK8-dependent [79]. However, these reports only examined CDK-mediated TAD serine phosphorylation of STATs under the context of canonical STAT activation in cancer. To date, little is known regarding whether CDKs are able to phosphorylate TAD serine residues of STATs in the absence of tyrosine phosphorylation (non-canonical STAT activation) or whether they are also involved in regulation of IFN-related functions beyond the proliferation of cancer cells.

## 3. Function of STAT Family Members and Regulation of STAT Activation

### 3.1. Function of STAT Family Members

In addition to be activated by IFNs, STAT1 also responds to other cytokines. Studies from gain-of-function mutations suggest that increased and prolonged phosphorylation of STAT1 is observed in response to IL-6 and IL-21 [80]. STAT2 appears unable to bind to DNA directly [81,82], but contributes a potent transactivation as a component of ISGF3. This complex recruits additional co-factors, such as p300/CBP, GCN5, and DRIP150, to initiate gene expression [81,82]. STAT2 can form alternative complexes with IRF9 without STAT1, which is different from the canonical IFN-α signaling [82,83].

STAT3 was initially identified as an IL-6-dependent transcription factor that promotes acute phase gene expression [84]. It is now known that STAT3 transduces signals for the entire IL-6 family (IL-6, IL-11, IL-31, LIF, CNTF, CLC/CLF, NP, CT1, OSM) and the IL-10 family (IL-10, IL-19, IL-20, IL-22, IL-24, IL-26), as well as granulocyte colony stimulating factor (G-CSF), leptin, IL-21, and IL-27 [85]. IL-6 is known largely for its role in induction of acute phase proteins [86]. It is interesting to note that the functions of IL-6 and IL-10 are diametrically opposite: IL-6 contributes to inflammation, whereas IL-10 blocks inflammation, even though both cytokines transmit signals through STAT3 [28]. Since the JAK/STAT interactions appear to be more complex than previously expected, it is possible that STAT3 is not the only STAT activated by IL-6 or IL-10. Ultimately, other STATs and their corresponding domain-negative isoforms may all influence the final outcome.

STAT3 has also been shown to be involved in mammary gland development. Lack of STAT3 causes a severe delay in post-lactational regression of the mammary gland, as observed in epithelium-specific STAT3-knockout mice [87]. However, unexpected suppression of apoptosis of epithelial cells was observed, as well, in STAT3 conditional knockout mice with delayed mammary gland involution [88], implying that STAT3 plays a role in cell transformation. Moreover, it has been demonstrated that STAT3 is constitutively active in some murine and human tumors, and regulates Src-dependent transformation of fibroblasts [89]. Indeed, constitutively phosphorylated STAT3 has served as a disease marker in a large number of breast cancer patients [90]. However, another study demonstrated that STAT3 controls lysosome-mediated cell death in the mammary gland during post-lactational regression in vivo [91]. These conflicting results indicate that STAT3 assumes complex roles depending on context and interactions among multiple factors. Meanwhile, some reports suggested an involvement of STAT3 in the IFN-mediated antiviral response as STAT3 is specifically required for induction of a subset of IFN-α driven ISGs [49,92]. These observations are also consistent with the antiviral function of oncostatin M (OSM), a member of the IL-6 family, which is demonstrated to induce an antiviral response via JAK/STAT3 signaling [93].

STAT4 was initially identified through screening for STAT homologues, and was found to share 52% identical amino acids with STAT1 [94]. Expression of STAT4 was found to be limited to myeloid cells, NK cells, dendritic cells, and T lymphocytes [94,95]. Subsequent studies showed that STAT4 is activated by IL-12, a cytokine that plays a critical role in the development of the Th1 subset of T helper cells by stimulating IFN-γ production and enhancing expression of T-box 21 (TBX21 or T-bet) [85,96,97]. Although it was initially thought that neither IFN-α nor IFN-γ could activate STAT4 [94], a later study revealed that activation of STAT4 by IFNs is species-specific [98]. In humans, IFN-α/β can drive Th1 development by activating STAT4 without IL-12-induced signaling, but the same phenomenon is not observed in mouse [98]. Further study demonstrated that IFN-α induced STAT4 activation requires the presence of activated STAT2 [99]. However, instead of being directly recruited to the IFN-α receptor complex, STAT4 is indirectly recruited by a mechanism involving STAT2 [99]. Although both Th1 and Th2 cells express STAT4, STAT4 is only activated by IL-12 in Th1 cells, since IL-12 receptors are absent from Th2 cells [100]. STAT4 has highly specific functions, as observed in STAT4-deficient mice. Although these mice exhibit normal total T cell counts, they exhibit Th1 defects and enhanced development of Th2 cells [101]. Meanwhile, STAT4 is required for cytolytic functions of NK cell [102].

Although STAT4 plays important roles in driving differentiation of T helper cells, its molecular mechanism of action is largely unknown. By using chromatin immunoprecipitation and high-throughput sequencing to compare the transcriptional profiles of STAT4 and STAT6, STAT4 was found to bind over 4000 genes with distinct binding motifs [103]. Among those 4000 genes, more than 2300 genes are specific targets of STAT4 and these genes may be involved in Th1 differentiation [103]. Meanwhile, STAT4 plays a more dominant role in promoting active epigenetic marks [103]. Moreover, a recent study demonstrated that STAT4 deficiency in mice causes a failure to induce lung Th2 or Th17 immunity upon RSV challenge, but enhances the lung RSV-specific CD8+ T cell response to secondary RSV challenge [104].

STAT5A and STAT5B were found to be encoded by two linked genes, *STAT5a* and *STAT5b* [85]. These two proteins share 96% identity and are only divergent at their carboxyl termini. STAT5 was originally identified as mammary gland factor (MGF), which is the central mediator of the lactogenic hormone response in mammary epithelial cells [105]. In addition to its role as a prolactin-activated transcription factor, STAT5 proteins are activated by various cytokines and other factors, including members of the IL-3 family (IL-3, IL-5, and GM-CSF), the IL-2 family (IL-2, IL-7, TSLP, IL-9, IL-15, and IL-21), growth hormone (GH), Epo (erythropoietin) and Tpo (thrombopoietin) [85]. Although STAT5A and STAT5B display functional redundancy due to their structural and functional similarities, STAT5A single knockout mice are predominately defective in prolactin (RPL)-dependent mammary gland development, while STAT5B single knockout mice exhibit defects similar to those observed in GH receptor-deficient mice [85]. Meanwhile, STAT5A and STAT5B double knockout mice demonstrated that STAT5 is necessary for T cell proliferation and generation of NK cells [106], as well as IL-2-mediated signaling [107]. STAT5 can also act as an oncogene, and was found to be constitutively phosphorylated in cancer cells, especially in some myeloid leukemias [108,109]. It has been demonstrated that STAT5 can be phosphorylated constitutively by oncogenic tyrosine kinases, such as fusion tyrosine kinases (FTKs) generated by chromosomal translocations [110]. Moreover, two isoforms of STAT5A have been identified: wild-type STAT5A (794 amino acids) and a deletion mutant expressing truncated STAT5A (772 amino acids); the latter lacks the C-terminal transactivation domain [34], and this domain-negative isoform is generated by specific proteolytic processing, not by RNA splicing [35]. In addition, a recent report shows that STAT5B is a dominant player in both effector and regulatory (Treg) responses, suggesting that it is necessary for immunological tolerance [111]. Analyses of genomic distribution and transcriptomic output indicate that STAT5B has great impact on gene expression, but its relative abundance determines functional specificity.

STAT6 was originally identified from cellular extracts as an IL-4-stimulated STAT, and was soon shown to be activated by IL-13 as well [112,113]. STAT6 plays an important role in regulating acquired immunity involving IL-4 secretion by activated T and B lymphocytes, mast cells, and basophils, whereby STAT6 promotes activation of several cell types, most notably, Th2 cells [85]. In STAT6-deficient mice, IL-4-induced signaling in lymphocytes is impaired, and is unable to induce upregulation of class II MHC expression and CD23, and consequently exhibit impaired immunoglobulin isotype switching [114,115]. Data gained from chromatin immunoprecipitation studies demonstrate that STAT6 binds over 4000 genes, with more than 2000 genes shared with STAT4 [103]. However, the molecular basis for STAT6 function is still largely unknown. Meanwhile, it has been demonstrated that STAT6 regulates lung inflammatory responses in animal models [116]. In this role, STAT6 has been found to contribute to alternative activation of macrophages and lung antiviral responses in a JAK-independent manner [117].

### 3.2. Regulation of the JAK/STAT Pathway

Since STAT proteins function as essential mediators of cytokine- or hormone-induced signaling to promote cell development, proliferation, and differentiation, activation of STATs is tightly regulated. Suppressive regulators of STAT activity include protein inhibitors of activated STAT (PIAS) family, suppressors of cytokine signaling (SOCS), and ubiquitin carboxy-terminal hydrolase 18 (USP18) [118,119].

In mammals, PIAS proteins are encoded by four genes: PIAS1, PIAS2 (also known as PIASx), PIAS3, and PIAS4 (also known as PIASy) [120]. Except for PIAS1, each PIAS gene encodes two isoforms. Functionally, PIAS family proteins are currently known as Small Ubiquitin-like Modifier (SUMO) E3 ligases [121,122,123] and all PIAS family members appear to regulate STAT signaling [120]. PIAS1, PIAS3, and PIAS2 have been shown to inhibit STATs activation via interactions with STAT1, STAT3, and STAT4, respectively [124,125,126], while STAT1 also interact with PIAS4 [127]. PIAS proteins negatively regulate activation of STATs after binding to them. However, PIAS proteins only interact with the STAT dimer, which indicates that only phosphorylated STATs can interact with PIAS [128]. In addition to STATs, PIAS members also affect functions of other transcription factors by acting as SUMO E3 ligases [129]. Meanwhile, SUMOylation of STATs by PIAS has been identified as a modulatory mechanism as well [130,131,132,133]. It was suggested that SUMOylation of STAT1 obstructs phosphorylation of the proximal tyrosine residue, leading to semiphosphorylated STAT dimers which compete with their fully phosphorylated counterparts and interfere with activation of the JAK/STAT pathway [132].

The suppressor of cytokine signaling (SOCS) family is another group of negative regulators for the JAK/STAT pathway [134]. The SOCS family of mammalian hosts is comprised of eight members, SOCS1 to 7, and CIS (cytokine-induced SH2 domain-containing protein) [135]. All SOCS proteins share a common structure containing an SH2 domain and a C-terminal SOCS box domain [135]. The SOCS box domain is critical for proteasome-mediated degradation of SOCS-associated proteins [135]. However, SOCS1 and SOCS3 contain an kinase inhibitory region (KIR) that inhibits kinase activity of JAKs [119]. Therefore, SOCS family members inhibit JAK/STAT via different mechanisms, such as blocking of STAT recruitment to cytokine receptors, targeting of STATs for proteasome degradation, binding to JAKs, and targeting JAKs for proteasome degradation [136,137,138]. SOCS1 and SOCS3 are the major inhibitors for type I IFN-mediated signaling [139]. SOCS1 inhibits IFN signaling via interaction with TYK2 [140], while SOCS3 binds to JAK2 in a similar manner via its KIR to inhibit IFN signaling as well [141].

USP18, a protein of 368 aa in length and an ISG15 isopeptidase, is a negative regulator of type I and III IFN-activated JAK/STAT signaling [142], and is rapidly upregulated by viral infection and IFNs. Absence of USP18 strengthens IFN signaling, whereby the inhibitory role of USP18 is independent of its activity as an isopeptidase. Moreover, USP18 inhibits JAK/STAT signaling via specific binding of IFNAR2 to block JAK1 interaction with IFNAR2 and downstream signaling [143].

Besides the well-defined inhibitors described above, JAK/STAT signaling can also be regulated by cysteine-based protein tyrosine phosphatases (PTPs), which dephosphorylate pTyr residues in the JAK/TYK activation loop or phosphorylation sites within cytoplasmic domains of cytokine receptors [144]. However, the detailed mechanism of PTPs regulation of the JAK/STAT pathway is still unclear.

In addition to SUMOylation of STATs by PIAS, post-translation modification (PTM) has been shown to regulate STATs activation as revealed by proteomic technologies. Modification of certain sites among STATs can result in either positive or negative effects on STATs activation [145]. Acetylation has been detected of STAT1, STAT2, STAT3, STAT5b, and STAT6, and is reviewed elsewhere [146]. STAT acetylation is dependent on the balance between histone deacetylases (HDACs) and histone acetyltransferases (HATs), such as CBP/p300 [146]. Generally, acetylation of STATs increases their DNA binding affinity and promotes transcriptional activation, protein–protein interaction, and STAT dimerization. Moreover, acetylation of STATs can occur at various lysine residues located within different domains that include the DNA-binding, SH2, N-terminal, and C-terminal domains [146,147]. Furthermore, it is interesting that SUMOylation and acetylation can involve the same lysine residue in STAT5 lysine 696 (K696), although not concurrently [148,149]. This finding therefore suggests that SUMOylation and acetylation might maintain a balance in STATs function.

Another mechanism for regulating STAT activation involves both arginine- and lysine-based methylation of STATs [150,151]. The role of STAT methylation is complicated, as it exhibits both negative and positive roles during STAT activation. As the first identified arginine methylation site in STAT1, arginine 31 (R31) methylation was shown to be required for transcriptional activation [152]. However, a later study reported that inhibition of STAT1 methylation at R31 results in prolonged half-life of STAT1 tyrosine phosphorylation [150], and thus, R31 methylation negatively regulates STAT1 activation. However, methylation at R27 of STAT6 is necessary for optimal STAT6 phosphorylation, nuclear translocation, and DNA-binding activity, all of which have effects distinct from those reported for STAT1 [153]. Recently, a new methylation site in STAT1 (K525) has been identified, and is required for STAT1-mediated antiviral immunity [154]. Moreover, STAT3 could be reversibly methylated at K140 and K180 by histone methyltransferases SET9 and EZH2, respectively [66,155]. In addition, mass spectroscopy analysis reveals that unphosphorylated-STAT3 (U-STAT3) is acetylated at K685, and that K685 integrity is required for expression of most U-STAT3-dependent genes [156].

Besides methylation and acetylation, ISGylation involving covalent bonding of targets to interferon-stimulated gene 15 (ISG15, a ubiquitin-like protein) has been shown to regulate IFN signaling as well [157,158]. An earlier study revealed that mice lacking UBP43, a protease that removes ISG15 from conjugated targets, are hypersensitive to type I IFN. Furthermore, in UBP43-deficient cells, IFN induced prolonged STAT1 tyrosine phosphorylation [159]. A recent study has suggested that ISGylation of STAT1 increases stability of STAT1 and prevents premature termination of the immune response in LPS-stimulated microglia [160]. Therefore, it appears that both in vitro and in vivo data indicate that ISGylation is a positive regulator of IFN signaling.

Collectively, PTM of STATs represents a novel mechanism for JAK/STAT regulation. However, more studies are needed to understand this regulatory mechanism, since crosstalk between methylation, SUMOylation, and acetylation is still not understood. Moreover, a recent study demonstrated that inhibition of HDAC enhances STAT acetylation but blocks NF-κB signaling during renal inflammation and fibrosis in haplotype Npr1+/− male mice [161]. Therefore, crosstalk between the JAK/STAT and NF-κB pathways under the same PTM conditions is complicated, and needs further study. On the contrary, although dysregulation of STAT PTM during virus infection has been reported as an important viral tactic to evade the antiviral response mediated by IFNs [162,163], there has been little investigational focus on this issue, to learn whether virus infection could affect PTM of STATs to regulate the JAK/STAT pathway.

### 3.3. Virus-Induced Serine Monophosphorylation of STATs and Inflammatory Responses during Virus Infection

Since the discovery of virus-encoded IFN antagonists, it has been proposed that JAK/STAT pathway antagonism is a virulence factor that might be exploited to achieve virus attenuation during vaccine development. This speculation is further supported by the observation that mice lacking intact JAK/STAT signaling machinery (IFN receptor or STAT1 knockout mice) are highly susceptible to virus infection [21,22,23,24]. Meanwhile, in vivo data suggest that a rapid type I interferon response protects astrocytes from flavivirus (tick-borne encephalitis virus, Japanese encephalitis virus (JEV), West Niles virus (WNV), and Zika virus (ZIKV)) infection and virus-induced cytopathic effects [164]. Antagonism of JAK/STAT signaling by both DNA and RNA viruses has been extensively reviewed by Fleming [165]. It appears that almost all viruses examined so far encode antagonists for the JAK/STAT pathways, as well as for IFN induction or NF-κB-mediated signaling [19]. However, one mystery that cannot be fully explained regarding the interaction between virus and host signaling is how virus induces an inflammatory response while concurrently generating antagonists of both upstream and downstream IFN signaling (or of other cytokines) within the JAK/STAT axis.

As a typical example, porcine reproductive and respiratory syndrome virus (PRRSV) is known for its ability to inhibit both IFN induction and IFN-activated JAK/STAT signaling, with several PRRSV antagonists of JAK/STATs previously identified [52,93,166,167]. For detail about PRRSV mediated antagonism for IFN induction and signaling, please see reviews by Nan et al. [168]. For a long time, the antagonism for both IFN induction and IFN activated signaling by PRRSV was considered to contribute PRRSV virulence and pathogenesis [168]. However, in vivo studies comparing the pathogenesis of high-pathogenic PRRSV strain (HP-PRRSV, the most virulent type of PRRSV), classical PRRSV strain (less virulent than HP-PRRSV) and attenuated vaccine strain of PRRSV tell a different story. It has been demonstrated that rapid replication of HP-PRRSV in pigs could trigger aberrant sustained expression of pro-inflammatory cytokines and chemokines, leading to a robust inflammatory response that is likely to contribute to virulence of HP-PRRSV [169]. Meanwhile, in another in vivo study comparing HP-PRRSV-HuN4 (a strain belongs to HP-PRRSV) to its homologous vaccine strain HuN4-F-112 (attenuated strain by serial passage of HP-PRRSV-HuN4 in tissue culture) [170], the HP-PRRSV-HuN4 strain generated earlier and higher levels of inflammatory cytokines [171]. This result indicates that HP-PRRSV-HuN4 may enhance inflammation to cause more damage to tissues and organs. However, the attenuated HuN4-F112 vaccine strain induced lower levels of inflammatory cytokines that enhanced immune responses against infection [171]. Therefore, it appears that IFN-JAK/STAT axis antagonist genes identified within the PRRSV virus genome during in vitro studies are not consistent with the observed phenotypes difference of PRRSV in vivo.

As discussed in the previous section, monophosphorylation of serine residues in STATs has been frequently reported as non-canonical TAD serine phosphorylation in the absence of tyrosine phosphorylation [41]. This observation may imply novel functions of STATs during virus infection and pathogenesis. Except for the identification of JAK/STAT pathway antagonists within the PRRSV genome, our previous research demonstrated that PRRSV infection promotes IFN-independent serine monophosphorylation of STAT1 (S727) via non-structure protein (nsp) 12, resulting in higher levels of pro-inflammatory cytokines expression in vitro [74]. Meanwhile, it is notable that PRRSV-induced serine monophosphorylation of STAT1 (S727) and cytokine expression could be blocked by SB203580, a MAP kinase-specific inhibitor. Taken together, these results suggest that generation of virus-induced pSTAT1-S727 depends on the p38 MAP kinase pathway [74].

As mentioned above, HP-PRRSV infection in pigs is characterized by aberrant expression of pro-inflammatory cytokines and chemokines, leading to a robust inflammatory response [169,171]. Our data also demonstrated that PRRSV-induced serine monophosphorylation of STAT1 appears to be linked to PRRSV virulence, since the MLV vaccine has a minimal effect on induction of pSTAT1-S727 in vitro, while the heterogeneous virulent strain VR2385 strongly induces serine monophosphorylation of STAT1 (S727) and expression of pro-inflammatory cytokines [74]. Although these lines of evidence require further investigation, these in vitro data are consistent with in vivo observations of phenotypic differences in inflammatory responses between HP-PRRSV strain (HP-PRRSV-HuN4) and attenuated homologous vaccine strain (HP-PRRSV-HuN4-F-112) [169,171]. Therefore, it appears that expression of pro-inflammatory cytokines and chemokines promoted by IFN-independent monophosphorylation of STAT1 comprise an alternative explanation for the cytokine storm observed during HP-PRRSV infection.

In addition to PRRSV, virus-induced serine monophosphorylation of STAT1 has been reported for other DNA and RNA viruses, such as Epstein–Barr virus (EBV) and human immunodeficiency virus-1 (HIV-1), as well [172,173,174]. Although EBV was shown to inhibit IFN activated JAK/STAT pathways via a variety of mechanisms [175,176], abnormal activation of STAT1 and STAT3 (as defined by nuclear translocation and binding with DNA by STAT proteins) was observed very earlier in EBV-related lymphoma cell lines, as well as samples from patients with Burkitt’s Lymphoma (a malignant neoplasm of the haemopoietic system associated with EBV) [177]. In fact, EBV was the first virus reported to induce serine monophosphorylation of STAT1 during infection, and serine monophosphorylated STAT1 is able to bind DNA in EBV-infected cells [172]. However, this earlier study did not offer a clear linkage between DNA-bound STAT1 (serine monophosphorylation), and evaluated expression of genes normally responsive to STAT1 [172]. Instead, the authors postulated that serine monophosphorylated STAT1 could be employed by EBV as an alternate strategy to override the antiviral response evoked by IFNs. In their scenario, competition between serine monophosphorylated STAT1 and IFN-stimulated STAT1 for DNA-binding sites would block IFN-activated JAK/STAT signaling [172]. In another study, it had been shown that an early lytic nuclear protein encoded by EBV, the SM protein, is capable of specifically promoting the expression of several ISGs that are known to be strongly induced by IFN in the absence of IFN induction or JAK/STAT activation, except through the induction of STAT1 expression [174]. This is interesting because several mechanisms of interference of JAK/STAT1 activation by IFN have been proposed for EBV, such as activation of SOCS3 to dampen JAK/STAT signaling [178], or the use of virus-encoded miRNA (BART16) to target the CREB-binding protein [179]. Therefore, it appears that EBV is capable to block IFN-induced canonical JAK/STAT activation and stimulate ISG expression in an IFN-independent manner. These observations mirror a similar scenario as observed for PRRSV. Ultimately, EBV-induced serine monophosphorylated STAT1 might be the key to explaining these controversial observations.

Besides EBV, participation of STATs in viral pathogenic responses was also observed in human immunodeficiency virus (HIV)-1 as well. The Tat protein of HIV-1, a regulatory protein for viral transcription enhancement, contributes to the immune evasion of HIV by inducing SOCS3 expression to antagonize IFN-induced canonical STAT activation [180]. However, HIV-1 infection of the central nervous system leads to HIV-1 encephalitis (HIVE), which is fueled by viral infection and immune activation of brain mononuclear phagocytes (MPs: blood-derived perivascular macrophages and microglia) [181]. It was believed that HIV-1 activates pro-inflammatory and IFN-inducible genes in human brain microvascular endothelial cells (HBMECs) and is a contributing factor to HIVE [182]. A later study confirmed that serine monophosphorylated STAT1 and STAT3 were both observed in HIV-1-infected HBMECs and correlated with HIV-1-induced inflammatory responses and neuropathogenesis [173]. By contrast, a specific STAT1 inhibitor, fludarabine (FLUD), blocked HIV-1 induced serine monophosphorylation of STAT1, and thus decreased IL-6 expression and secretion [173]. It is also notable that direct exposure of HBMECs to HIV-1 virion induced phosphorylation of STAT1 and STAT3 at S727 [173]. However, the tyrosine phosphorylation of STAT1 and STAT3, as well as serine phosphorylation of STAT-2, STAT-5, or STAT-6, were not observed [173]. These data suggested that serine monophosphorylated STAT1 and STAT3 might serve as contributing factors to HIV-1-induced inflammatory responses in HBMECs.

Meanwhile, studies on Kaposi’s sarcoma-associated herpesvirus (KSHV) demonstrated that latent protein kaposin B promotes serine monophosphorylation of STAT3 at S727 in the absence of tyrosine phosphorylation at Y705 [183]. KSHV infection in human umbilical vein endothelial cells (HUVECs) induces consecutive phosphorylation of STAT3 at serine 727 while phosphorylation of STAT3 at tyrosine 705 is transient [183]. After transduction of HUVECs via retroviruses encoding kaposin B, HUVECs expressing kaposin B protein harbored increased pSTAT3 S727 in the nucleus, while tyrosine phosphorylation is absent for these STAT3 [183]. Unlike STAT1, evidence suggests that serine monophosphorylation of STAT3 at S727 is activated by MAP kinase-activated protein kinase 2 (MAP2K2) [183]. However, serine monophosphorylated STAT3 still leads to elevation of STAT3-dependent genes, including CCL5, IL-6, and transforming growth factor β (TGF-β) [183]. This observation is consistent with proposed function of KSHV kaposin B, which is linked with evaluated inflammatory cytokine level during KS pathogenesis [184]. Therefore, it appears that serine monophosphorylated STAT3 induced by either KSHV or HIV-1 is linked with increased inflammatory cytokine responses akin to those observed for serine monophosphorylated STAT1 during PRRSV infection [74,173,183].

So far, serine monophosphorylation induced by virus infection has been only reported for STAT1 and STAT3. Although it is still unknown if other STATs could have monophosphorylation of serine residues at TAD domains, neither tyrosine phosphorylation nor serine phosphorylation of STAT2, STAT5, and STAT6 was observed in HBMECs exposed to HIV-1 virion. [173]. Therefore, it is also notable that available data implies a correlation between viral-induced serine monophosphorylation of STATs (STAT1 and STAT3) and pro-inflammatory responses caused by virus infection. It is interesting that most virus-encoded antagonists block canonical JAK/STAT activation, while some viruses are still capable of inducing expression of ISGs or other inflammation responses both in vitro and in vivo. However, it remains unknown whether virus-induced serine monophosphorylation is common to all STATs, or if it is restricted to certain STATs, such as STAT1 or STAT3, since canonical activation of STAT1 and STAT3 trigger pro-inflammatory responses. Meanwhile, it would be meaningful to study whether unphosphorylated ISGF3 (U-ISGF3) harbors serine monophosphorylated STAT1 or completely unphosphorylated STAT1 (lacking both tyrosine and serine phosphorylation), since previous reports of U-ISGF3 only focused on tyrosine phosphorylation without examining serine phosphorylation [54,55]. Taken together, the correlation of non-canonical STATs activation, serine monophosphorylated STATs, and unphosphorylated STATs during viral infection needs further investigation. Such studies may yield information insightful to understanding viral pathogenesis, especially regarding virus-induced cytokine storms and inflammation-related immune pathogenesis.

## 4. Conclusions and Perspectives

Since induction and signaling pathways of IFNs are well-defined, and great progress has been made to understand virus antagonism of the IFN-JAK/STAT axis, many questions regarding the IFN-JAK/STAT axis remain unanswered. Although IFN types and subtypes (type I and type III) appear to be redundant in their functions due to the activation of common downstream JAK/STAT pathway, the exact differences between those IFN subtypes remain unknown. Indeed, such distinctions may be an artifact of an arbitrarily simple classification of IFNs that overlooks the functionally heterogeneous nature of IFN types and subtypes. Additionally, since the antiviral functions of IFNs rely on ISGs, elucidation of ISG functions is also of interest, as most ISGs are not well-defined. Notably, some ISGs also respond to other transcription factors, such as members of the IRF family, activator protein 1 (AP-1), and NF-κB [2,185]. Therefore, crosstalk between the JAK/STAT pathway and other signaling pathways merits further study.

Meanwhile, the roles of IFN-activated JAK/STAT signaling in viral virulence and pathogenesis also require further investigation. This is especially true with regard to the in vivo role that IFN antagonists play, since most of these viral antagonists have been identified using in vitro screening methods. Moreover, crosstalk between virus-mediated antagonism of canonical JAK/STAT activation, viral-induced activation of non-canonical JAK/STAT pathway, and inflammatory responses promoted by viral infection may be also linked to viral pathogenesis. In the coming decades, additional mechanisms likely will be revealed to further our understanding of the connection between non-canonical JAK/STAT activation and inflammation-linked viral pathogenesis.
